# A case of Marfan syndrome with massive haemoptysis from collaterals of the lateral thoracic artery

**DOI:** 10.1186/s12890-019-1033-1

**Published:** 2020-01-08

**Authors:** Yuki Yabuuchi, Hitomi Goto, Mizu Nonaka, Hiroaki Tachi, Tatsuya Akiyama, Naoki Arai, Hiroaki Ishikawa, Kentaro Hyodo, Kenji Nemoto, Yukiko Miura, Isano Hase, Shingo Usui, Shuji Oh-ishi, Kenji Hayashihara, Takefumi Saito, Tatsuya Chonan

**Affiliations:** 1 Department of Respiratory Medicine, National Hospital Organization, Ibaraki Higashi National Hospital, 825, Terunuma, Tokai-mura, Ibaraki, Naka-gun 319-1113 Japan; 2 Department of Clinical Research, National Hospital Organization, Ibaraki Higashi National Hospital, Ibaraki, Japan; 3 Department of Medicine, Nikko Memorial Hospital, Ibaraki, Japan

**Keywords:** Giant pulmonary cysts, Chronic intrathoracic inflammation, Non-bronchial systemic arteries

## Abstract

**Background:**

Marfan Syndrome (MFS) is a heritable connective tissue disorder with a high degree of clinical variability including respiratory diseases; a rare case of MFS with massive intrathoracic bleeding has been reported recently.

**Case presentation:**

A 32-year-old man who had been diagnosed with MFS underwent a Bentall operation with artificial valve replacement for aortic dissection and regurgitation of an aortic valve in 2012. Warfarin was started postoperatively, and the dosage was gradually increased until 2017, when the patient was transported to our hospital due to sudden massive haemoptysis. Computed tomography (CT) with a maximum intensity projection (MIP) revealed several giant pulmonary cysts with fluid levels in the apex of the right lung with an abnormal vessel from the right subclavian artery. Transcatheter arterial embolization was performed with angiography and haemostasis was achieved, which suggested that the bleeding vessel was the lateral thoracic artery (LTA) branch. CT taken before the incident indicated thickening of the cystic wall adjacent to the thorax; therefore, it was postulated that the bleeding originated from fragile anastomoses between the LTA and pulmonary or bronchial arteries. It appears that the vessels exhibited inflammation that began postoperatively, which extended to the cysts.

**Conclusion:**

We experienced a case of MFS with massive haemoptysis from the right LTA. We have to be aware of the possibility that massive haemoptysis could be induced in MFS with inflamed pulmonary cysts.

## Background

It has been reported that 16% of patients with Marfan Syndrome (MFS) have pulmonary disease, and that these pulmonary diseases contribute to 10% of deaths in MFS [1, 2]. Pneumothorax in MFS arising from the labile wall of pulmonary cysts is well-known and one of the severe complications of MFS [1, 2]. On the other hand, a rare case of MFS with massive intrathoracic bleeding has recently been reported, which caused hemopneumothorax and haemoptysis [3, 4]. We report here a case of MFS with massive haemoptysis associated with pulmonary cysts; it was suggested that anastomoses between the branches of pulmonary and lateral thoracic artery (LTA) were the origin of the bleeding.

## Case presentation

The patient was a 32-year-old male ex-smoker. He was diagnosed as MFS at the age of 26 based on an aortic dissection (Stanford type A), aortic regurgitation and marfanoid habitus. A Bentall operation with an artificial valve replacement for aortic dissection and a regurgitation of the aortic valve was performed. Thereafter, he was required to take warfarin for the artificial heart valve and was followed at a local hospital. His adherence to medication was poor, which went unnoticed, resulting in the dose of warfarin gradually being increased to 6.5 mg per day in 2017, when he was admitted to the emergency unit of our hospital suffering from a massive haemoptysis and dyspnoea.

The patient had a history of left pneumothorax at the age of 16; however, the details of this are unknown since his mother died in his childhood and the father has been missing since then. On presentation, the patient was fully conscious with a height of 198 cm, body weight of 77 kg, blood pressure of 105/56 mmHg, pulse rate of 90 bpm, body temperature of 36.9 °C, and a saturation of percutaneous oxygen (SpO_2_) of 94% in room air. The physical examination revealed coarse crackles on auscultation, especially in the right upper lung areas. He had characteristic features such as a tall height, arachnodactyly, dolichocephaly and retrognathia, which are compatible with MFS.

**Fig. 1:**
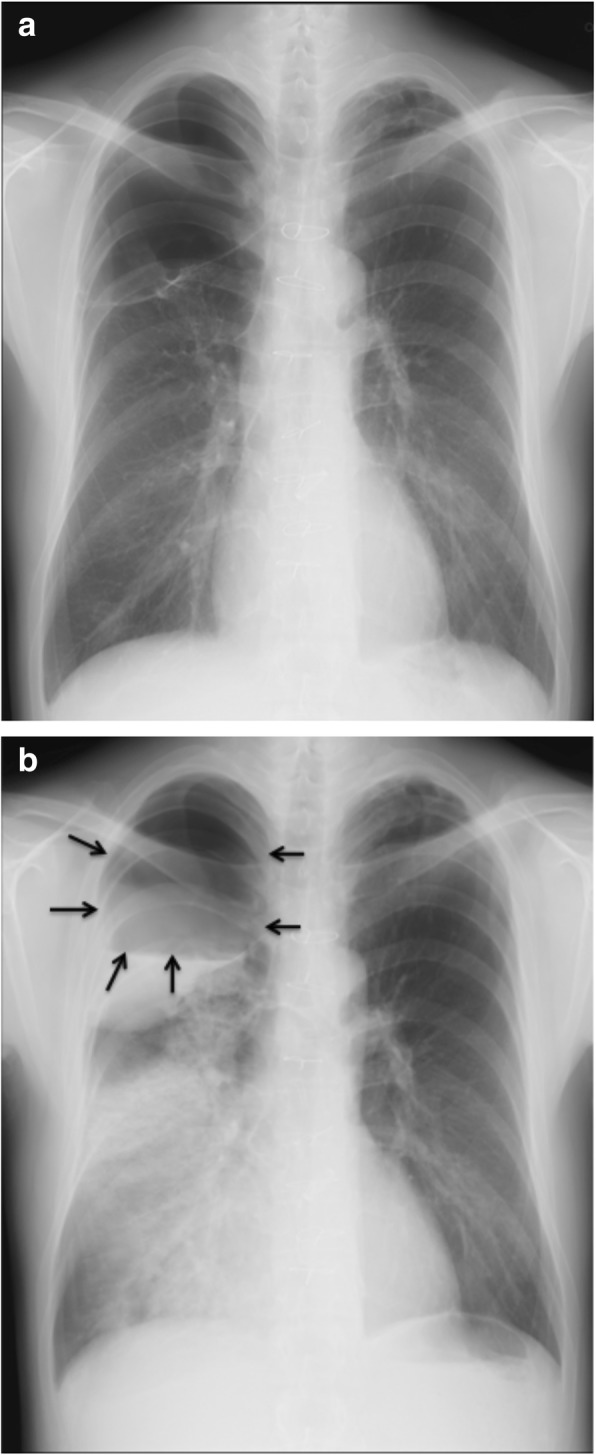
Chest X-ray showing an apical giant bleb in the right lung before a massive haemoptysis (**a**). There was an air fluid as an accumulation of blood in the bleb and a large consolidation in the middle and lower lung after bleeding (**b**). The arrows indicate the margin of the pulmonary cyst and its fluid level (**b**)

**Fig. 2:**
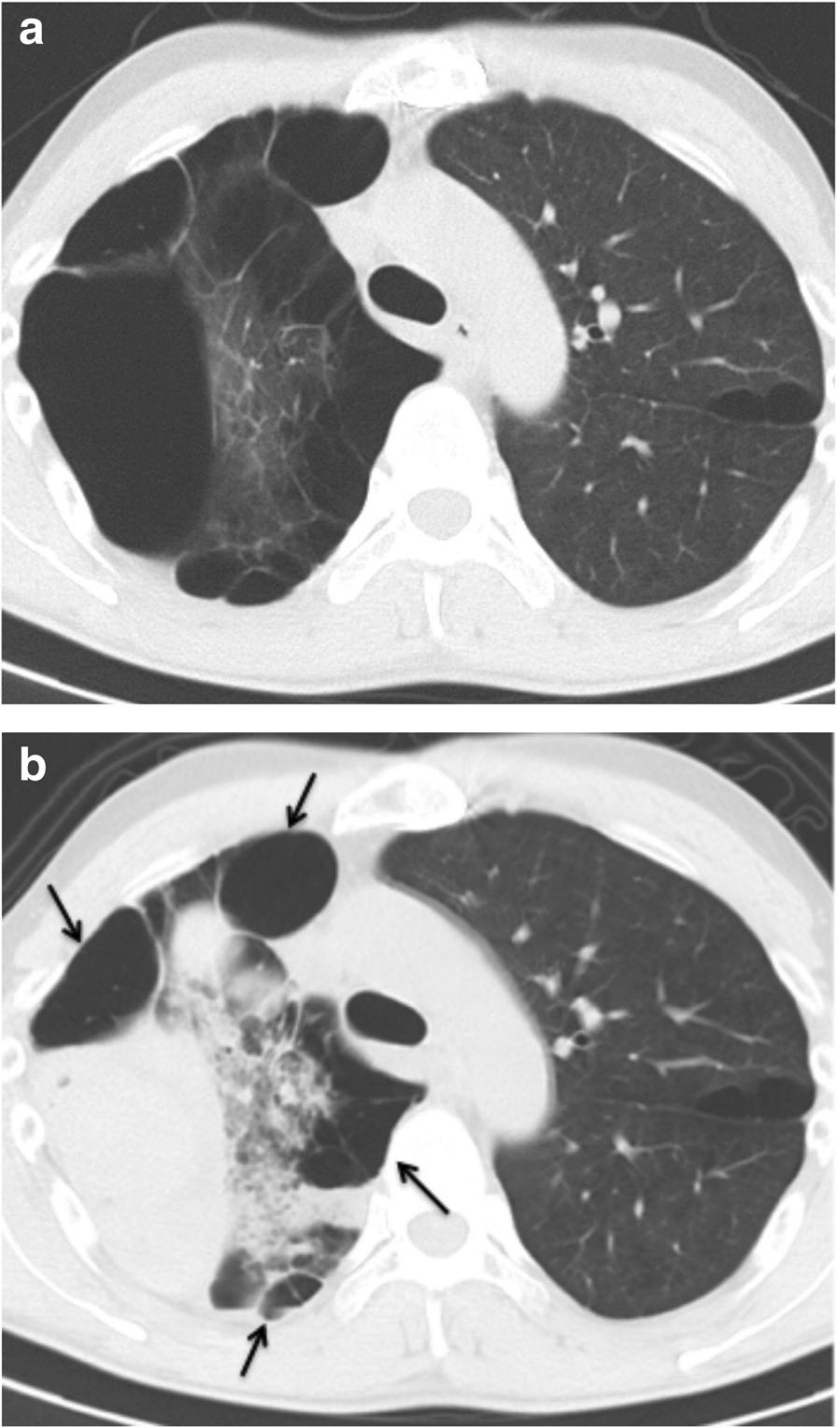
Images of chest computed tomography (CT) showing multiple cysts and thickening of the pleura around the cysts in the upper lobe before haemoptysis (**a**). There was a tumour shadow with a uniform density in the right upper lobe, which was demonstrated to be full of blood in the cyst (**b**). Another giant cyst adjacent to the tumour storing blood presented an air fluid image (**b**). The arrows indicate the margin of the multiple pulmonary cysts (**b**)

**Fig. 3:**
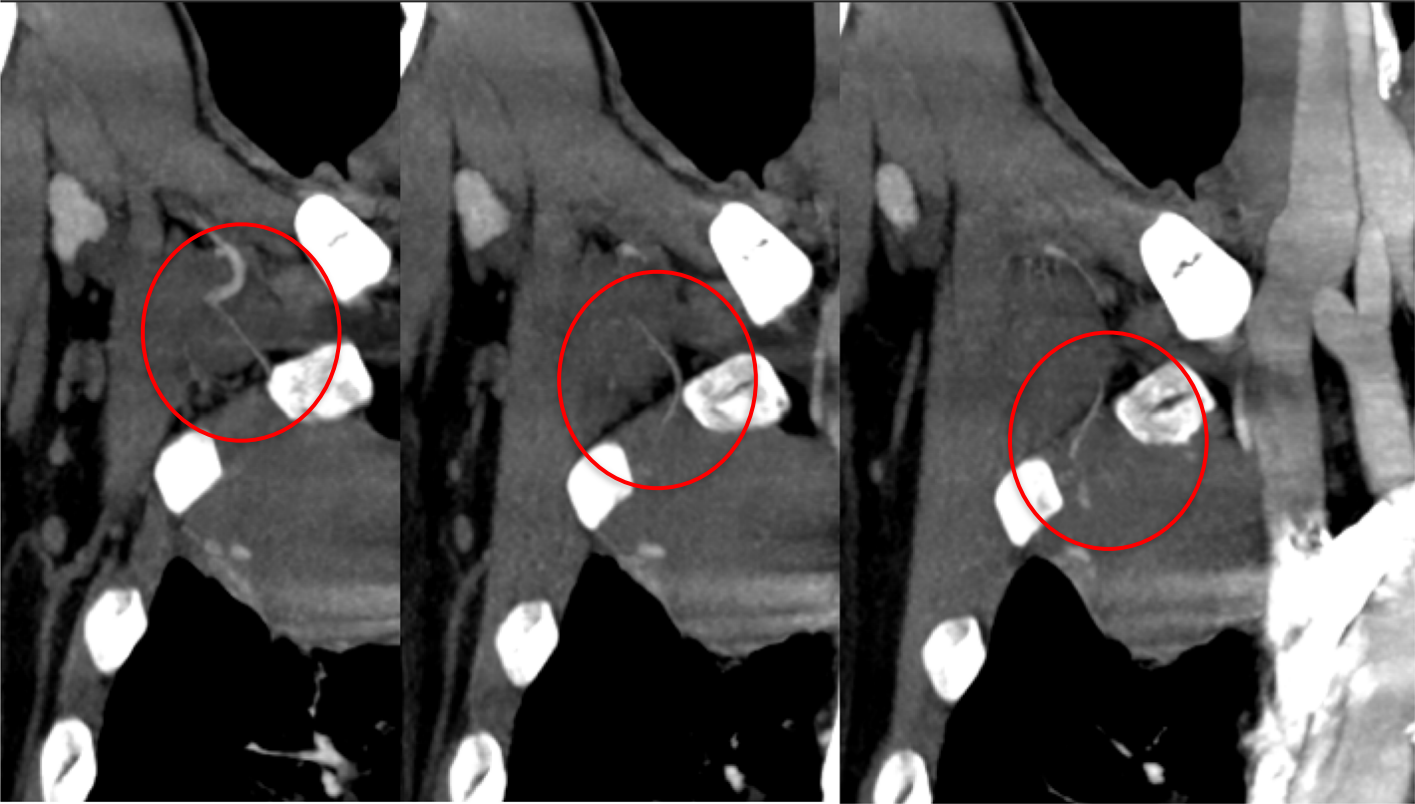
Coronal slice images of a maximum intensity projection (MIP) showing an abnormal vessel from the right subclavian artery extending into the cyst (Fig. 3). The vessel was later revealed by angiography to be the lateral thoracic artery. The apical giant cyst had pleural adhesions resulting from inflammation due to an invasive operation, which induced the vessel to grow abnormally

Laboratory data demonstrated anaemia (blood haemoglobin: 10.0 g/dL), an elevated white blood cell count (12,300 /μL), a prothrombin time of 32.5 s (international normalized ratio PT-INR: 5.21), and an active partial thromboplastin time of 61.2 s. Serum tumour markers, antibodies of connective tissue disease and markers of infection such as *Aspergillus* and *Mycobacterium* were all negative. Pathological bacteria were not detected in sputum culture. A chest X-ray showed a right apical pulmonary cyst with an air-fluid level inside and consolidation in the right lower lung field (Fig. 1b). A chest computed tomography (CT) scan indicated multiple cystic lesions in the upper lobes (Fig. 2b) and a maximum intensity projection (MIP) detected an abnormal vessel originating from the right subclavian artery, which extended into the cyst (Fig. 3).

**Fig. 4:**
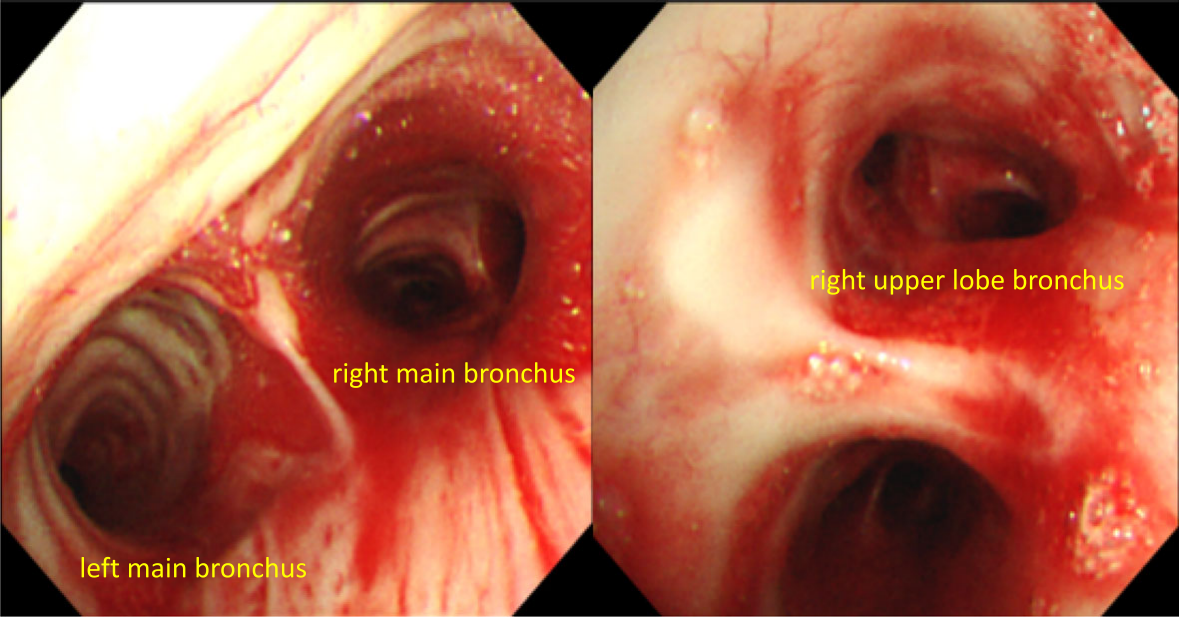
Bronchoscopic images showing bleeding from the right upper lobe bronchus (Fig. 4). A large amount of blood flowed into the right truncus intermedius and left main bronchus

**Fig. 5:**
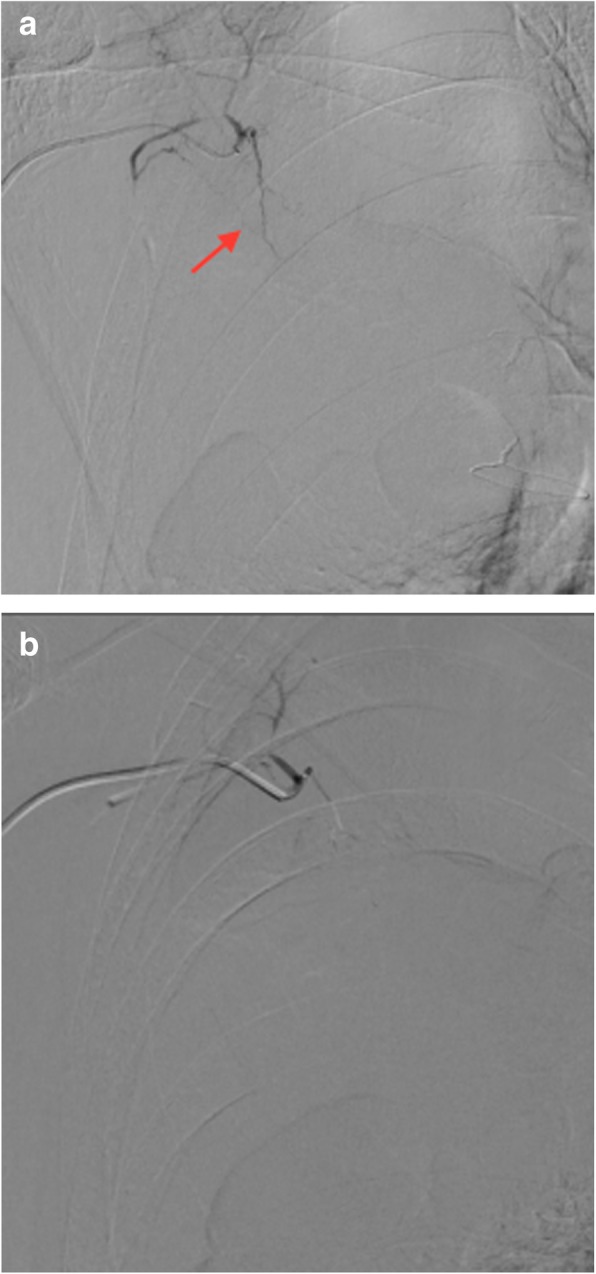
Angiography showing abnormal vessels from the lateral thoracic artery (LTA) into the right lung (**a**). The arrow indicates the abnormal vessels. Embolization of the LTA was performed (**b**), resulting in haemostasis

An emergency bronchoscopy revealed continuous bleeding from the apical segmental bronchus of the right lung (B1) (Fig. 4), which appeared to have been stopped by topical application of epinephrine and thrombin. Vitamin K and haemostatic medications were infused, which was followed by a transfusion of four units of blood. After emergent therapy, the patient was transferred to the cardiovascular unit of another hospital. An angiography was performed and the abnormal vessel was radiographically diagnosed as the LTA (Fig. 5a, b). Moreover, the bleeding was stopped successfully by embolization of the artery, which confirmed that the abnormal vessel responsible for bleeding was the LTA. The patient’s condition improved, and warfarin was restarted with strict control of PT-INR. The patient has since been stable with no signs of re-bleeding.

## Discussion and conclusion

A case of MFS with massive haemoptysis was presented; the bleeding plausibly originated from the extended collaterals of the right LTA. It is suggested that intrathoracic inflammation induced by surgical intervention together with fragile abnormal vessels due to MFS evoked the massive bleeding.

There have been two reports that have presented MFS with massive intrathoracic bleeding [3, 4]. In the present case, it is suggested that massive bleeding occurred on the basis of chronic inflammation of pulmonary cysts. It appears that pleural adhesions and inflamed cysts are common between the past case [3] and the present case, with the difference in the responsible arteries, i.e. the bronchial arteries in the former and the LTA in the latter case. This is the first case of MFS we are aware of that showed massive haemoptysis caused by collaterals of the LTA. It might be argued that the role of the collaterals was not confirmed pathologically. However, the bleeding was stopped by embolization of the LTA, which strongly suggests that bleeding occurred from collaterals of the LTA.

It has been reported that, in chronic inflammatory disorders, angiogenic growth factors are released from the inflammatory site, which promotes neovascularization and remodelling of pulmonary vessels, and hence facilitates anastomoses between the pulmonary and systemic circulations [5]. It has been reported that these collaterals, which connect the pulmonary and systemic circulations, mostly originate from bronchial arteries [6]. However, in the present case, the non-bronchial arteries were the counterpart of the pulmonary circulation [6]. Pulmonary cystic diseases such as bullae and blebs are often accompanied by chronic inflammation, which facilitates the proliferation of branches and anastomoses as compensation for decreased blood supply [7].

Arterial blood under increased systemic arterial pressure is prone to extravasation into the surrounding tissue, resulting in massive haemoptysis [5, 6]. In our case, the patient had previously had multiple thoracic surgeries on the numerous existing pulmonary cysts that were probably related to MFS. Pleural effusion occurred after these operations, which could facilitate chronic pleural inflammation and pleurodesis, as seen by CT scan (Fig. 2a). As a result, a collateral grew abnormally into the cyst and likely anastomosed with the pulmonary artery. Systemic arterial pressure from the lateral thoracic artery exerted pressure on the pulmonary circulation, which in turn induced the rupture of the vessel under the influence of an overdose of warfarin. Furthermore, the collaterals may have been prone to rupture due to the connective tissue disorder responsible for MFS.

We cannot completely rule out the possibility of mycotic infection, which could have induced an aneurysm. However, serum tests including *Aspergillus* precipitating antibody, *Aspergillus* galactomannan antigen and beta-glucan were all negative and fungi were not detected in cultured bloody phlegm obtained from the right upper lobe bronchus by bronchoscopy. Therefore, the origin of bleeding was unlikely to be related to a mycotic infection.

Here, a case of MFS was presented with massive haemoptysis, which occurred from the collaterals of the LTA. In the post-cardiac operative period, MFS patients need to be aware of collateral vessels in the lung.

## Data Availability

Not applicable.

## Abbreviations

B1: Apical segmental bronchus
CT: Computed tomography
LTA: Lateral thoracic artery
MFS: Marfan syndrome
MIP: Maximum intensity projection
PT-INR: Prothrombin time-international normalized ratio
SpO_2_: Percutaneous oxygen saturation
